# Plain to point network reduced graphene oxide - activated carbon composites decorated with platinum nanoparticles for urine glucose detection

**DOI:** 10.1038/srep21009

**Published:** 2016-02-15

**Authors:** Mohammad Faruk Hossain, Jae Y. Park

**Affiliations:** 1 Department of Electronic Engineering, Micro/Nano Devices & packaging Lab., Kwangwoon University, 447-1, Wolgye-Dong, Nowon Gu, Seoul, 139-701, Korea

## Abstract

In this study, a hydrothermal technique was applied to synthesize glucose-treated reduced graphene oxide-activated carbon (GRGO/AC) composites. Platinum nanoparticles (PtNP) were electrochemically deposited on the modified GRGO/AC surface, and chitosan-glucose oxidase (Chit-GOx) composites and nafion were integrated onto the modified surface of the working electrode to prepare a highly sensitive glucose sensor. The fabricated biosensor exhibited a good amperometric response to glucose in the detection range from 0.002 mM to 10 mM, with a sensitivity of 61.06 μA/mMcm^2^, a short response time (4 s) and a low detection limit of 2 μM (signal to noise ratio is 3). The glucose sensor exhibited a negligible response to interference and good stability. In addition, the glucose levels in human urine were tested in order to conduct a practical assessment of the proposed sensor, and the results indicate that the sensor had superior urine glucose recognition. These results thus demonstrate that the noble nano-structured electrode with a high surface area and electrocatalytic activity offers great promise for use in urine glucose sensing applications.

Enzymatic glucose biosensors are significant tools for use in environmental and food analysis as well as in pharmaceutical and human metabolism research. The sensing mechanism usually consists of the immobilization of enzymes onto suitably-modified electrode surfaces to provide highly selective, sensitive, and rapid analysis of biological species, including DNA, Human IgG, and glucose^1^,^2^,^3^. In particular, glucose oxidase (GOx) is an enzyme that is commonly used in the specific recognition of glucose. On the other hand, amperometric glucose biosensors based on GOx are simple and reliable, have a high selectivity, and can be produced at a low cost, so these are widely used to detect the concentration of glucose^4^,^5^,^6^. Although non-enzymatic glucose biosensors have obvious benefits when determining the glucose concentration, such biosensors are affected by poisons in the intermediates and therefore exhibit a short linear detection range and have poor selectivity.

Noble metal nanoparticles (NPs) can be loaded onto carbon materials to obtain a high catalytic activity and good chemical durability. Such materials exhibit the following advantages over macro/microelectrodes when used for electroanalysis: an improvement in the mass transport and catalysis, a high effective surface area, and control over the electrode microenvironment^7^. For instance, Pt and Au nanoparticles are very effective when used as a matrix for enzyme sensors since these are biocompatible and have a large surface area^8^,^9^. Moreover, metal nanoparticles provide electrochemical reversibility through redox reactions, which is not possible with bulk metals^10^. Electrodeposition (cyclic voltammetry) is the most suitable technique to synthesize metal NPs since it allows for the size, density and the shape of the NPs to be controlled by adjusting its parameters, including the potential, charge, deposition rate, concentration, and composition of the metal precursor solutions^11^. More recently, noble metallic micro/nanoparticles loaded onto carbon nanotubes and graphene based substrates have been reported to dramatically improve the sensor activity^12^.

Reduced graphene oxide (RGO) thin films are a promising alternative to those made from pristine graphene or CVD-grown graphene. RGO is a plain graphite monolayer packed into a two-dimensional honeycomb lattice that can be used in various biosensor applications. However, RGO in aqueous solution results in the aggregation of sheets due to its hydrophobic nature and produces strong *π-π* interactions when the oxygen-containing functional groups have been removed during the reduction process^13^.

Moreover, the RGO sheets form curled, folded, and corrugated structures on the substrate due to the flexibility of the sheets^14^. The large folds arise after drying the substrate^15^ whereas smaller waves tend to be an inherent feature of the structure of isolated layers^16^. As a result, the RGO exhibits a lower conductivity than that predicted from theory. In contrast, activated carbon (AC) is commonly used as the electrode material to produce the electrochemical field since it has a large specific surface area and a low production cost. However, the problem is that AC is aggregated after loading on the substrate, and as a consequence, a large fraction of carbon atoms cannot be accessed by the ions in the electrolyte. As a result, graphene oxide and activated carbon composites have been developed to effectively overcome the limitations of the use of individual material. For instance, RGO can provide a flexible bridge that forms a “plane-to-point” (RGO-to-AC) conducting network, which reduces the aggregation of AC particles as well as RGO sheets and improves the electron transfer within the composite electrode^17^. To the best of our knowledge, this composite that has been newly developed is shown to be suitable for use for biosensor applications.

In this study, a hydrothermal technique was used to develop glucose-treated reduced graphene oxide and activated carbon (GRGO-AC) composites, and rectangular-shaped electrodes were fabricated to easily cast the composite suspension and to avoid oxygen plasma etching within the three electrode gaps. PtNP was electrochemically deposited on the electrode modified with the GRGO-AC composite to increase the area of the electrochemical surface. Glucose oxidase-chitosan composite with nafion was immobilized onto the GRGO/AC/PtNP modified sensing electrode surface, and cyclic voltammograms and amperometric measurements were used to characterize the devices for use in glucose sensor applications.

## Results

### Design and fabrication of biosensor

**Figure 1:**
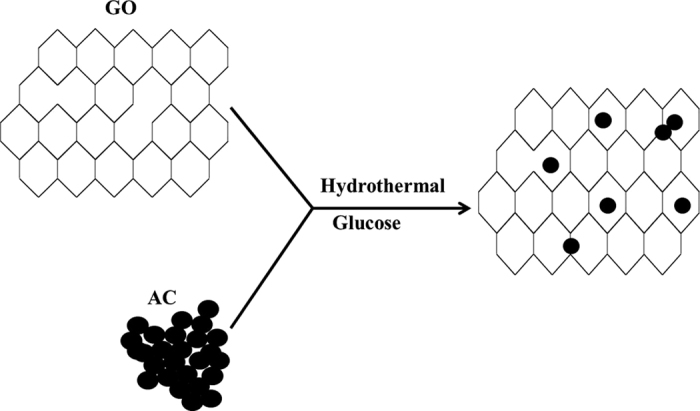
Illustration of the development process for the GRGO/AC composites.

**Figure 2:**
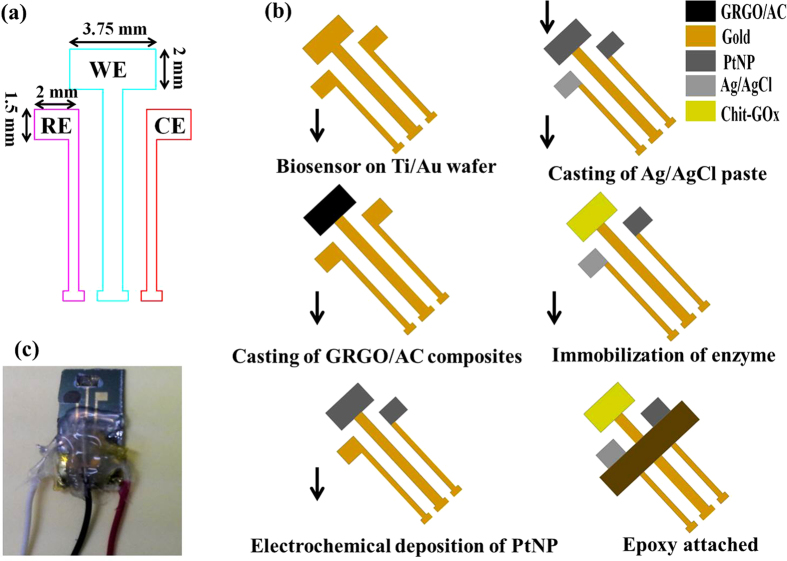
(**a**) Illustration and (**b**) fabrication sequences of the proposed glucose biosensor, (**c**) photomicrograph of the fabricated device.

The synthesis process of the GRGO/AC composites is illustrated in Fig. 1. Figure 2a shows a conceptual drawing of the rectangular-shaped enzymatic glucose biosensor. The biosensor was fabricated by photolithography technique. The enzymatic glucose biosensor was designed using three different electrodes consisting of a working electrode (GRGO/AC/PtNP/Chit-GOx/nafion), a counter electrode (PtNP), and a reference electrode (Ag/AgCl). The detailed preparation processes was described in the methods section. An illustration of the fabrication procedures and a photomicrograph of the enzymatic biosensor are shown in Fig. 2b,c, respectively.

### Physical characterization of the GRGO/AC composites

**Figure 3:**
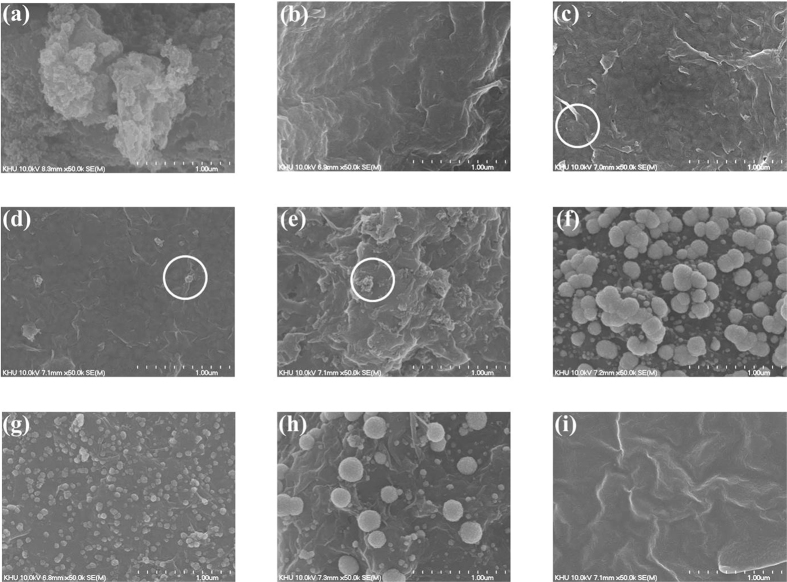
FESEM images of (**a**) AC, (**b**) GRGO, (**c**) GRGO/AC-12.5, (**d**) GRGO/AC-25, (**e**) GRGO/AC-50, (**f**) GRGO/AC-12.5/PtNP- 25 mV, (**g**) GRGO/AC- 12.5/PtNP-50 mV, (**h**) GRGO/AC-12.5/PtNP-100 mV and (**i**) GRGO/AC/PtNP/Chit-GOx/nafion.

The surface morphology of the as-prepared GRGO/AC composites was conducted via FESEM. The FESEM image of AC, formed after 2.5 h of ultrasonication in DMF and water, is shown in Fig. 3a. The AC particles are clearly shown to aggregate after drying on the substrate, and a given number of carbon atoms are not utilized effectively in terms activating their electrochemical function. Several layers of GRGO sheets formed on the Au surface, and the typical crumpled structure of the GRGO sheet that formed during the chemical reduction is shown in Fig. 3b. The chemical reduction was presumed to have caused the corrugation and rippling observed for GO. 12.5% of activated carbon was mixed with graphite oxide to form the GRGO/AC-12.5 composite sheet on the electrode, as shown in Fig. 3c. An increase of the AC particles into the composites results in an increase in the aggregation of the AC particles, which is clearly shown in the circles in Fig. 3d,e. FESEM images of the PtNP on GRGO/AC-12.5 composite sheets are shown in Fig. 3f–h with different scan rates. Cyclic voltammetry was carried out to deposit PtNP on the surfaces of the working electrodes modified with GRGO-AC composites. These figures show that the NPs have been decorated over the entire surface of the modified composites. Further NP deposition on the electrode surface is observed when the deposition time is higher (scan rate lower). In contrast, when the deposition time is lower, the NP deposition on the surface is also lower^18^ as shown in Fig. 3h,g. Chitosan and GOx were covalently bonded onto the GRGO/AC/PtNP surface. A nafion membrane was cast on the enzyme-immobilized surface to prevent leakage of the enzyme and to ensure the long term stability of the sensor, as shown in Fig. 3i. This figure clearly shows that the modified biosensor materials are tightly fixed on the surface of the electrode.

**Figure 4:**
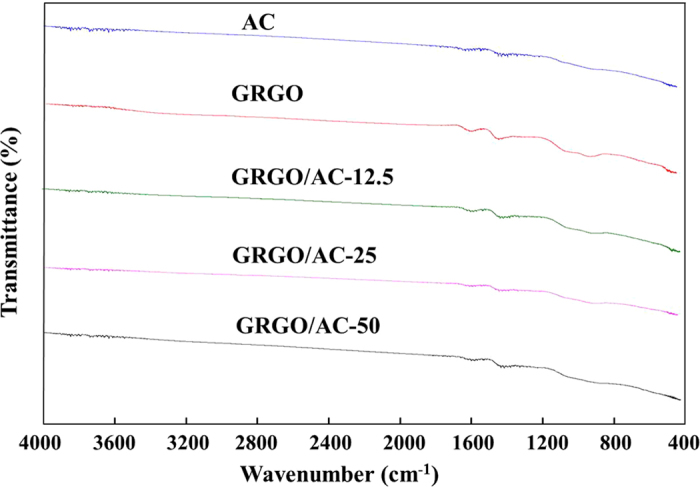
FTIR spectrum of AC, GRGO, GRGO/AC-12.5, GRGO/AC-25, GRGO/AC-50.

The Fourier transform infrared (FTIR) transmission spectra were obtained to investigate the surface groups present on AC, GRGO, and their developed composites. Figure 4 shows the FTIR spectra of the AC, GRGO and the developed composites. GRGO, AC and composite powders were used to collect the infrared spectra. A number of bonds contained in the GRGO, AC, and composites are clearly visible in these figures. The peak intensity of GRGO is also seen to be lower than that of AC and the developed composites. This result indicates that most of the bonds contained in the GRGO were removed during the reduction process. However, major peaks were found for GRGO at 3450.9 cm^−1^, 1704.5 cm^−1^, 1570.7 cm^−1^, and 1064.1 cm^−1^. The peak at 3450.9 cm^−1^ corresponds to the –OH stretching vibration while the peak at 1704.4 cm^−1^ corresponds to the C=O stretching vibration^19^. The peak at 1570.7 cm^−1^ for RGO is indexed to the C=C skeletal vibration of the graphene sheets^20^, which confirms the successful reduction of graphene oxide. A peak at 1057 cm^−1^ is attributed to the C-O in C-OH or C-O-C functional groups^21^. The major peaks for AC can be seen at 3460.89 cm^−1^, 1716.27 cm^−1^, 1506.50 cm^−1^, 1145.3 cm^−1^ and 637.8 cm^−1^. The peak at 3460.89 cm^−1^ is a result of the absorption of water molecules, which forms an O-H stretching mode for the hydroxyl groups and the adsorbed water. The peak at 1716.27 cm^−1^ is associated with the carbonyl group C=O, and the peak at 1506.50 cm^−1^ may be attributed to the aromatic carbon –carbon stretching vibration. A peak at 1057 cm^−1^ is attributed to C-O in C-OH or C-O-C functional groups. The C-C stretching vibration arises with a peak at 637.8 cm^−1^ 22,23. In addition, the peak at 1506.50 cm^−1^ may be attributed to the aromatic carbon–carbon stretching vibration.

The FTIR spectra of the developed composites clearly exhibit the major peaks for both GRGO and AC in the graph GRGO/AC-12.5, GRGO/AC-25 and GRGO/AC-50. The band spectra for a lower loading containing the developed composites are also seen to be pronounced in the band spectra of the matrix. The band spectra change with a gradual increase in the loading into the composites. Therefore, GRGO and AC composites are confirmed to have been made using the proposed method.

**Figure 5:**
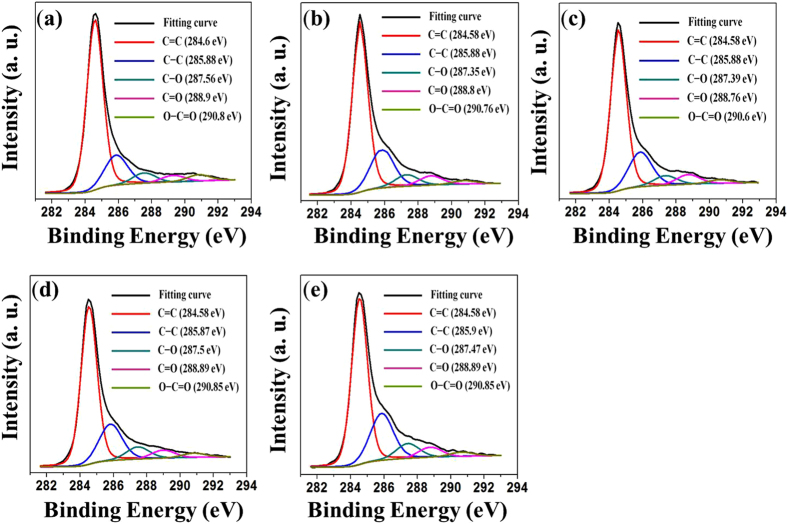
Typical C1s spectrum of (**a**) AC, (**b**) GRGO, (**c**) GRGO/AC-12.5, (**d**) GRGO/AC-25, (**e**) GRGO/AC-50.

An XPS analysis was carried out to determine the functional groups that were contained in the AC, GRGO and the developed composites. The typical C1s spectra for the AC, GRGO, and the developed composites are displayed in Fig. 5a–e. An analysis of results for AC shows that there are five carbon states in the C1s region, as shown in Fig. 5a. Five different peaks indicate a considerable degree of oxidation that corresponds to carbon atoms in different functional groups. The peaks are centered at binding energies of 284.6 eV, 285.88 eV, 287.56 eV, 288.9 eV and 290.7 eV. The peak at 286.6 eV corresponds to the non-oxygenated ring C included in C=C bonds to make sp^2^ hybridized carbon. Another peak (285.88 eV) is from sp^3^ hybridized carbon and includes the C-C bond. The C in C–O bonds (287.56 eV) that include hydroxyl and epoxy groups, the C in C=O bonds (288.9 eV) that include carbonyl groups and the C in O-C=O bonds (290.8 eV) incorporate carbolic acids or ester groups. The peak positions of these functional groups have been described well in the literature^24^. However, the peak positions may shift by small amounts relative to those indicated in the literature due to the chemical nature of the neighboring atoms on an individual surface. Five peaks were also found in the XPS curves of GRGO. The peak positions are at binding energies of 284.58 eV, 285.88 eV, 287.35 eV, 288.8 eV and 290.76 eV in Fig. 5b. The functional groups of AC and GRGO are almost indistinguishable, which indicates that the raw carbon material was activated with an oxidation reagent^23^. In addition, the intensity in the peaks of the oxygen functional group significantly decreased during the reduction process^25^,^26^, which indicates that reduced graphene oxide formed under the given conditions. The curves obtained from the XPS analysis of the developed composites are shown in Fig. 5c–e. Figure 5c shows the C 1s spectra of the GRGO/AC-12.5 composite, and it contains five major peaks centered at 284.58 eV, 285.88 eV, 287.39 eV, 288.76 eV and 290. 6 eV, which correspond to different functional group bonds. The peaks are similar to the AC and GRGO found in the XPS analyses, and this result confirms that the GRGO and AC produce composites under the given conditions. The intensity of the peaks reduced as the AC loading in the composites increased, and this is clearly visible in Fig. 5d,e. This result indicates that as AC loading increases, matrix mismatching also increases in the composites. Therefore, water molecules as a byproduct of the reduction reaction are replaced in surface of the composites^27^.

### Device characterization for glucose detection

**Figure 6:**
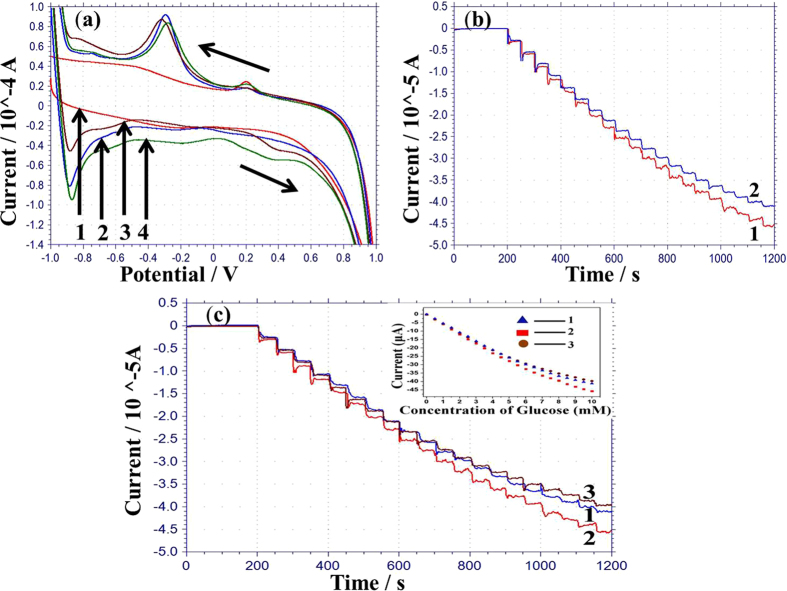
(**a**) Cyclic voltammograms (CVs) of the developed electrodes Au/GRGO/AC-12.5 (Curve 1), Au/GRGO/AC-12.5/PtNP (Curve 2), Au/GRGO/AC-12.5/PtNP/Chit-GOx/nafion in PBS only (Curve 3) and in 0.05 M PBS with 2 mM glucose (Curve 4), (PBS solution pH 7. 4, 0.05 M), scan rate: 50 mV/s. (**b**) Amperometric response of GRGO/AC-12.5PtNP/Chit-GOx/nafion (Curve 1) and GRGO/AC-25PtNP/Chit-GOx/nafion (Curve 2) based biosensors in PBS to the successive injection of a concentration of glucose in 0.5 mM at 0.3 V. (**c**) Amperometric response of GRGO/AC-12.5PtNP/Chit-GOx/nafion-based biosensors in PBS to the successive injection of a concentration of glucose in 0.5 mM at 0.2 V (Curve 1), 0.3 V (Curve 2) and 0.4 V (Curve 3) with inset calibration curves.

Figure 6a shows the typical cyclic voltammetry (CV) profile of the working electrode modified with the GRGO/AC-12.5 composite in 0.05 M PBS solution. GRGO-AC composites were used due to the good electron propagation within the GRGO-AC sheet and the substrate electrode. In addition, the carbon material is used on the substrate as a mediator for the enzymatic sensor^28^. The CV of the GRGO/AC-12.5 composite modified with PtNP at a 50 mV/s scan rate is shown Curve 1 in Fig. 6a. This figure shows that there is one strong oxidation peak and one reduction peak. The strong anodic peak at −0.85 V corresponds to the oxidation of Pt. Our previous study used Ag/AgCl with (3M NaCl) reference electrode, and an oxidation peak was observed at −0.6 V in PBS solution^29^. In this study, Ag/AgCl paste was used as a reference electrode. Therefore, a shift in the negative direction of 0.25 V may be seen relative to the Ag/AgCl (3M NaCl) reference electrode. The peak on the reduction curve at −0.3 V is related to the reduction of platinum oxide into platinum. After immobilizing the enzyme-chitosan with nafion on the working electrode, the peaks of the CV curve were reduced, as seen Curve 2 in Fig. 6a. This result indicates that glucose oxidase/nafion produces a barrier layer against electron transportation. In addition, there is no change in the CV curves after modification with enzymes and nafion. These results reveal that a hydrophobic protein layer can form on the surface of the oxidase-chitosan with nafion, which insulates the conductive support and the interfacial electron transfer after loading the glucose on the electrode^30^. 2 mM glucose was added in PBS, and the background oxidation current of the enzyme-chitosan/nafion-modified working electrode increased from −0.9 V to 0.7 V (Curve 4 in Fig. 6a). This may be a result of the increase in the amount of OH^-^ adsorbed on the surface of the working electrode. This result indicates that the composites and the PtNP-modified enzymatic electrode has a good electrocatalytic performance via glucose oxidation.

Figure 6b shows the current-time response for the hybrid enzymatic working electrode modified with GRGO/AC-12.5/PtNP (Curve 1) and GRGO/AC-25/PtNP (Curve 2) at 0.3 V upon the successive additions of 0.5 mM glucose. The GRGO/AC-12.5/PtNP-modified biosensor was subjected to amperometric measurements at 0.2 V (Curve 1), 0.3 V (Curve 2), and 0.4 V (Curve 3) upon a successive injection of 0.5 mM glucose in PBS solution, and the respective calibration curve is shown inside of Fig. 6c. The sensitivity of the developed biosensor is in the detection range from 0 to 10 mM with sensitivity of 55.05 μA/mMcm^2^, 61.06 μA/mMcm^2^, and 52.17 μA/mMcm^2^, respectively, at 0.2 V (Curve 1), 0.3 V (Curve 2) and 0.4 V (Curve 3). The highest sensitivity of the biosensor was observed for 0.3 V at 61.06 μA/mMcm^2^. This result indicates that glucose may be more oxidized at this potential as a biosensor surface. The sensitivity is higher than that reported in other studies with Au/RGO/PtPdNPs/GOx with a sensitivity of 24 μA/mMcm^2^ 29, PET/Ti/Au/SDS-MWCNT/PDDA/GOx/PDDA with a sensitivity of 5.6 μA/mMcm^2^ 31, Pt/TiO_2_/RGO/ PtNPs/GOx with a sensitivity of 0.94 μA/mMcm^2^ 32.

### Interference effect and biological sample test on biosensor

**Figure 7:**
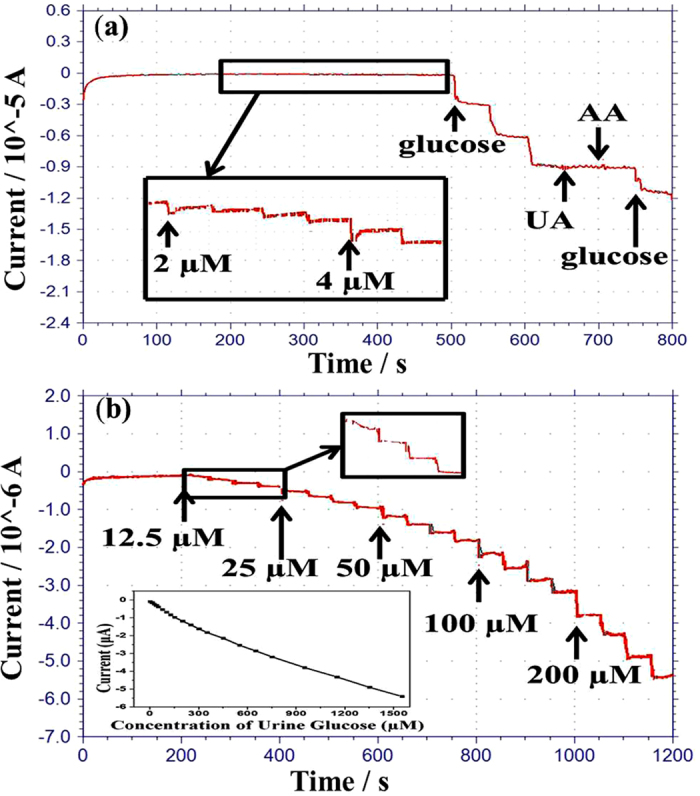
(**a**) Amperometric response of the interference effect on the biosensor upon the addition of 1.66 mM glucose, then 0.3 mM uric acid, 0.1 mM ascorbic acid (AA), and a further addition of 0.5 mM glucose in PBS solution (pH, 7.4, 0.05 M) at 0.3 V, with inset showing the detection limit. (**b**) Amperometric response of biosensor in PBS to the continuous injection of the different concentration of urine glucose at 0.3 V with inset calibration curves.

The selectivity of the glucose biosensor is important to avoid interference from other species. Ascorbic acid (AA), and uric acid (UA) are common interference species in biological samples, and the amperometric response of the fabricated glucose sensor was investigated for both of these. The oxidation current for the interference species (0.1 mM) was negligible when compared to the value of 1 mM glucose (5.93 μA), as shown in Fig. 7a. The biosensor was tested with a lower detection range of glucose. The detection limit of the biosensor was found to be 2 μM (the signal to noise ratio is 3), which is clearly shown in the inset of Fig. 7a. The detection limit is lower than that of other reported works like GCE/RGO/PAMAM/AgNP/GOx/Chit with a detection limit of 4.5 μM^33^, Cu/Cu_2_O/CuO/GOx with a detection limit of 5 μM^34^, and Pt/MWCNT/GOx a detection limit of 30 μM^35^.

The stability of the fabricated sensor was also observed for a period of 5 weeks. The glucose sensitivity decreased by 10.59% during that time, and these results indicate that the sensor has a long-term stability.

The capability of biosensor was investigated for practical applications by testing for glucose in human urine. Measuring glucose in human urine is considered to be a valuable, non-invasive method to monitor the state of diabetic patients when compared to testing glucose in human blood^36^. Urinary glucose levels of people with diabetes are often within the range from 2.8 to 5.6 mM^37^, and the normal range is from 0 to 0.8 mM. Therefore, a simple, reliable, cost-effective method to monitor glucose in urine is highly desired in order to better diagnose diabetes. In this study, a urine sample was collected from a healthy woman and glucose was not seen in the urine sample. Figure 7b shows the current response of the biosensor with successive injection of different concentration of urine glucose at 0.3 V and inset the calibration curve. It is seen that the biosensor responses very quickly and reaches a steady state within very a few seconds even though a low concentration of urinary sample was added. Furthermore, 100 μL of the urine sample were added into 20 mL of 0.05 M PBS solution. Then, standard glucose sample was added in urine and PBS diluted solution so that the concentration of glucose in urine sample could be detected from the calibration curve. The results are listed in Table 1, and the recovery of spiked glucose in the urine sample was observed in the range from 95 to 102% and RSD ranged from 4.1% to 5.6%, which indicates that the proposed biosensor has good ability for glucose detection in the human urine.

## Discussion

The AC particles are clearly shown to have occupied the region between the sheets of the GRGO network, as marked with a circle (Fig. 3c). The mean plane to point network provides good conduction pathways and electron transfer kinetics^17^. When the loading of AC particles increases, the amount of mismatch also starts to increase between the matrices in the composites. Large-scale loading was used in the composites because clear physical and chemical properties were observed. The morphological studies of the composites indicated that loading 12.5% and 25% of AC particles results in an effective distribution on the GRGO plane sheets.

Uniformly distribution of nanoparticles on the electrode surface is a key factor for enzyme immobilization. In particular, PtNP is uniformly distributed at a 50 mV scan rate, as seen in Fig. 3g. This result indicates that the electrochemical process was confined to the electrode surface^38^. A fairly smooth and fine dispersion of NPs provides a good platform for biosensing, and these characteristics indicate that the electrode is suitable as a platform for enzymatic sensors.

Clear information of composites was found from FTIR and XPS analyses data. An analysis of the XPS data shows that a low loading of AC based on the GRGO composites is better than that for a higher loading of the composites. The biosensor was thus optimized according to the electrochemical response of the electrode modified with the composites for glucose sensing.

The sensitivity of the GRGO/AC-12.5/PtNP and GRGO/AC-25/PtNP hybrid modified enzymatic biosensor was calculated to be 61.06 μA/mMcm^2^ and 54.55 μA/mMcm^2^, respectively, for a detection range from 0 to 10 mM (Curve 1 and Curve 2 in Fig. 6b). The response time for the GRGO/AC-12.5/PtNP and GRGO/AC-25/PtNP-based biosensors were 4 s and 6 s, respectively. The likely reason for this result may be the uniform, well-controlled distribution of the AC and GRGO in the composites (GRGO/AC-12.5), which can be clearly seen in the FESEM images. Thus, the amperometric response of the biosensors was investigated for the GRGO/AC-12.5/PtNP modified biosensor for more detailed experiments. The GRGO/AC-12.5-based biosensor also exhibited detection limit of 0.002 mM, good stability and acceptable interference. Urine glucose was tested to investigate the suitability of the biosensor for clinical applications. The sensor showed acceptable performance in detecting glucose in human urine.

In conclusion, an enzymatic glucose biosensor with rectangular-shaped electrodes was designed and fabricated on a single chip. A hydrothermal technique was used to successfully develop glucose-treated reduced graphene oxide and activated carbon (GRGO/AC) composites, and the composite suspensions were decorated on the working electrodes. Then, PtNP and enzyme-chitosan composites with nafion were placed on the on the modified biosensor working electrodes. The electrochemical data proves that the fabricated biosensor is a good candidate for use in the routine detection of glucose for diabetic patients.

## Methods

### Chemicals

Potassium hexachloroplatinate (K_2_PtCl_6_), graphite powder (44 μm size), ascorbic acid (AA), uric acid (UA), β-D(+) glucose, and hydrogen peroxide (30%), chitosan (white mushroom, higher molecular weight), nafion (5%), activated carbon, N-hydroxysuccinimide (NHS) and N-(3-dimethylaminopropyl)-N- ethylcarbodiimidehydrochloride (EDC) were purchased from Aldrich Co. (St. Louis, USA). The stock β-D(+) glucose (99.5%, Sigma) solution was prepared by diluting it in 0.05 M PBS (pH 7.4) solution, and all other solutions were prepared with deionized water (resistivity ≥18 MΩ-cm).

### Synthesis of the GRGO/AC composites

Graphite oxide was prepared using a modified Hummer’s method^39^. Then, 45.5 mg of graphite oxide and 6.5 mg of activated carbon were mixed into 26 mL of DI water, followed by ultrasonication for about half an hour (h). After that, the as-prepared mixture solution and the 0.1 M glucose were mixed, and this mixture was kept for 1 h. Next, the mixture was sealed in a teflon-lined autoclave and was maintained at 180 °C for 2 h in a convection oven. When the autoclave had cooled, the as-prepared gel was dispersed again into 1 M acetic acid aqueous solution and was left for 5 h. Finally, the mixture was washed several times with doubly-distilled water until reaching a pH of 7, and it was then dried overnight in an oven at 90 °C under a vacuum. The dried platelet is denoted as GRGO/AC-12.5. Similarly, GRGO/AC-25 and GRGO/AC-50 composites were prepared using 12.5 mg and 26 mg AC, respectively. Plain GRGO was also prepared in a similar manner, but without using activated carbon.

### Modification of the individual electrode

The sensor was fabricated A 30-nm titanium (Ti) layer was formed on top of a Si/SiO_2_ substrate via sputtering to provide good adhesion, and a diffusion barrier of gold (Au) and Si and a 150-nm Au layer provided a low resistivity for the overall system. 1 mg of the GRGO/AC composite platelet was dispersed into 1 mL of solution of dimethylformamide (DMF) and doubly distilled water (1:1) using an ultrasonicator for 2.5 h. Then, the mixture was manually stirred for a few minutes before casting. Finally, 6 μL of the as-prepared GRGO/AC suspension was dropped onto the working electrode of the biosensor and was dried in ambient conditions. The Ag/AgCl on the reference electrode was prepared by casting an Ag/AgCl paste.

The electrochemical deposition of PtNP was conducted using a three-electrode system. The GRGO/AC working electrode and a counter electrode were modified with PtNP by using a cyclic voltammogram with a potential ranging from −0.2 V to 0.7 V in a deaerated precursor solution consisting of 1.25 mM of K_2_PtCl_6_ and 60 mM H_2_SO_4_ for 10 cycles and 14 cycles, respectively. The nanoparticles were controlled on the working electrode by changing the scan rate. Then, these electrodes were rinsed with distilled water and were dried using nitrogen gas. The GRGO/AC containing a carboxylic group was activated using an NHS/EDC mixer with EDC as the coupling agent and NHS as the activator. 5 μL of 20 mM EDC and 20 mM NHS mixing solution were cast on the top of the modified working electrode, followed by incubation for 7 h at room temperature. Then, 5 mg of GOx and 3 mg of chitosan were dissolved into 0.5 mL of DI water (pH 6) and were then ultrasonicated for 5 min and stirred until casting. Afterwards, 5 μL of the mixture were cast on the surface of the working electrode modified with the coupling agent. Then, the solvent was allowed to dry in a refrigerator at 4 °C. Finally, 3 μL of nafion, ethanol and DI water solution (1:7:1) were dropped on the as-prepared working electrode and were allowed to dry for 10–15 min at room temperature. The modified working electrode was stored at 4 °C for 24 h, and the biosensor electrodes were stored in a refrigerator at 4 °C when not in use.

### Materials and electrodes characterization

The working electrodes morphologies were characterized by field emission scanning electron microscopy (FESEM; Hitachi S-4700). The surface groups present on AC, GRGO, and their developed composites were analyzed by FTIR (Thermo Nicolet Corp., Madison, WI) and the chemical compositions and status of AC, GRGO, and their developed composites were analyzed by XPS (ULVAC-PHI PHI-5000).

### Electrochemical characterization

Electrochemical deposition was conducted using a three-electrode system with an electrochemical analyzer (Model 600D series, CH Instruments Inc., USA). An Ag/AgCl with 3 mM NaCl and a flat Pt bar electrode were utilized as the reference and counter electrodes, respectively. Electrochemical characterization of biosensors was conducted by electrochemical analyzer using three electrodes system. Ag/AgCl paste and PtNP on the Au electrodes were acted as the reference and counter electrodes, respectively.

## Additional Information

**How to cite this article**: Hossain, M. F. and Park, J. Y. Plain to point network reduced graphene oxide - activated carbon composites decorated with platinum nanoparticles for urine glucose detection. *Sci. Rep.* **6**, 21009; doi: 10.1038/srep21009 (2016).

## Figures and Tables

**Table 1:** Determination of glucose in a human urine sample (n = 5).

Sample	Spiked (μM)	Detected (μM)	Recovery (%)	RSD (%)
1	25	24.5	98.0	4.1
2	75	75.2	102	5.3
3	200	190	95.0	3.9
4	2000	1977.5	98.87	5.6
